# Influence of Processing Parameters and Natural Antimicrobial on *Alicyclobacillus acidoterrestris* and *Clostridium pasteurianum* Using Response Surface Methodology

**DOI:** 10.3390/foods11071063

**Published:** 2022-04-06

**Authors:** Jasmine Hadj Saadoun, Alessia Levante, Martina Marrella, Valentina Bernini, Erasmo Neviani, Camilla Lazzi

**Affiliations:** Department of Food and Drug, University of Parma, Parco Area delle Scienze 49/A, 43124 Parma, Italy; jasmine.hadjsaadoun@unipr.it (J.H.S.); martina.marrella@unipr.it (M.M.); valentina.bernini@unipr.it (V.B.); erasmo.neviani@unipr.it (E.N.); camilla.lazzi@unipr.it (C.L.)

**Keywords:** antimicrobial, spore, response surface methodology, germination, inactivation

## Abstract

The food industry must ensure the stability of the products, and this is often achieved by exposing foods to heat treatments that are able to ensure the absence of pathogenic or spoilage microorganisms. These treatments are different in terms of temperature and duration and could lead to a loss in nutritional and sensory value. Moreover, some types of microorganisms manage to survive these treatments thanks to the sporification process. The addition of antimicrobials can become necessary, but at present, consumers are more inclined toward natural products, avoiding synthetic and chemical additives. Antimicrobials from plants could be a valuable option and, in this context, a patent concerning an antimicrobial extract from fermented plant substrate was recently tested against foodborne pathogens revealing high antimicrobial activity. The objective of this study was the creation of a model for the evaluation and subsequent prediction of the combined effect of different process and product variables, including antimicrobial addition, on the inhibition and reduction of spore germination of target microorganisms, *Alicyclobacillus acidoterrestris* and *Clostridium pasteurianum*, responsible for spoilage of tomato-based products.

## 1. Introduction

Tomato (*Solanum lycopersicum* L.) is one of the most cultivated vegetables in the world, and the global production accounts for around 170 million tons, where China is the largest producer, followed by the United States and India [1]. In Italy, about 5.5 million tons are intended for processing in the food industry each year [2]. Throughout the supply chain, from harvesting to the final product, large quantities are discarded. These can be either tomatoes that are not compliant for sale because they do not meet quality or appearance criteria, or by-products generated during the processing of the main product. One of the objectives of the European Union is to become a resource-efficient economy, by the adoption of a circular economy [3]. In this perspective, the reduction or repurposing of waste and by-products is promoted. In the tomato industry, peels and seeds represent about 10–30% of the by-products that are generated during processing [4]. These can be used as animal feed or for the recovery of bioactive components such as pectin, oil, fiber and lycopene [5]. A patent was recently registered by the University of Parma (n. 102019000006815), based on a lactic acid fermentation process of tomato peels and seeds and subsequent extraction, to obtain an extract with antimicrobial activity for food applications [6]. Its use has been tested on meat products and it is effective against pathogenic microorganisms belonging to *Salmonella* spp., *Listeria monocytogenes*, *Escherichia coli*, *Staphyloccocus aureus*, *Bacillus cereus* and *Pseudomonas* spp. [7].

Given the nature of the waste, and with a view to a circular economy, the use of this antimicrobial in tomato-based products should be evaluated to facilitate the recovery of resources lost during production. Moreover, considering the nature of the antimicrobial products, this finds space in the growing demand from consumers for natural preservatives [8].

In processed products, the food must be subjected to heat treatments that are able to ensure the absence of pathogenic or spoilage microorganisms. These treatments, depending on the product, are different in terms of temperature and time. However, some microorganisms manage to survive these treatments thanks to the sporulation process, activated when conditions are unfavorable and allowing dormancy for long periods. Therefore, spore-forming microorganisms can be found in tomato-based products causing off-flavors as principal defects. Among them are *Alicyclobacillus acidoterrestris*, which produces guaiacol that has a medicinal odor, or *Clostridium pasteurianum*, which can cause swelling of the package [9,10].

Different factors can influence microbial behavior: the pH of the medium, the heat treatment time to which they are subjected, or the concentration of antimicrobials, if present in the product. To understand the effect of different factors on growth or inactivation, experimental designs were developed and used in applied microbiology studies to evaluate different treatments and processing and using different target microorganisms [11,12,13,14].

The objective of this study was the building of a model for the evaluation and subsequent prediction of the combined effect of different process and product variables on the microbiological stability of possible tomato-based formulations. In particular, we evaluated the effectiveness of the patent antimicrobial n. 102019000006815 on the inhibition and reduction of spore germination of *Alicyclobacillus acidoterrestris* and *Clostridium pasteurianum* that often contaminate tomato-based products.

The approach proposed allowed us to identify the combination of optimal parameters for a reduction of these microbial targets in heat treatment. Specifically, for each microbial target, an experimental design that considered the effect of five factors on the inactivation and germination capacity of the spores was set up. These factors were identified in heat treatment time, water activity (a_w_), pH, antimicrobial concentration and spore concentration.

## 2. Materials and Methods

### 2.1. Alicyclobacillus acidoterrestris LMG 16906

*Alicyclobacillus acidoterrestris* was one of the target microorganisms, and the strain LMG 16906 was purchased from the Belgian Coordinated Collection of Microorganisms (BCCM/LMG) in freeze-dried form. The culture was revitalized three times with 3% (*v*/*v*) inoculum in the Alicyclobacillus acidoterrestris Medium (AAM) and incubated at 45 °C for 24 h. AAM is composed by (i) CaCl_2_·7H_2_O, 0.25 g; MgSO_4_·7H_2_O, 0.5 g; (NH_4_)_2_SO_4_, 0.2 g; yeast extract, 2 g; glucose, 5 g; KH_2_PO_4_, 3 g; and distilled water, 1 L, adjusted to pH 4.0 with H_2_SO_4_; (ii) 1 mL of trace elements solution (CaCl_2_·2H_2_O, 0.66 g; ZnSO_4_·7H_2_O, 0.18 g; CuSO_4_·5H_2_O, 0.16 g; MnSO_4_·4H_2_O, 0.15 g; CoCl_2_·6H_2_O, 0.18 g; H_3_BO_3_, 0.1 g; Na_2_MoO_4_·2H_2_O, 0.03 g; distilled water, 1 L). 

Spores were obtained by optimizing some protocols present in the literature [15,16,17]. Briefly, after revitalization *Alicyclobacillus acidoterrestris* LMG 16906 was grown for seven days in AAM broth at 45 °C. One hundred microliters of the culture broth were inoculated on the surface of the modified AAM agar medium, prepared by adding 50 mg/L of manganese chloride tetrahydrate to AAM, and incubated at a suboptimal temperature of 43 °C for 10 days ± 1. The presence of spores with respect to the vegetative cells was periodically checked using an optical microscope (Olympus System Microscope Models BX51, Tokyo, Japan), and when it reached about 90%, the spores were collected. An aliquot of Ringer (Oxoid, Basingstoke, UK) was inoculated onto the plates and by gently scraping the spore solution was collected, centrifuged (4000 rpm—20 min—4 °C) three times by suspending in Ringer and then stored at −20 °C. 

The sporal titer (Log cfu/mL) of the solution was evaluated by plate count following a thermal shock carried out at 80 °C for 10 min to facilitate the activation of the spores and the inactivation of any vegetative forms. The spore solution was decimal diluted sterile water with 0.1% peptone, dilutions were plated in AAM agar medium and plates were incubated at 45 °C for 48 h.

### 2.2. Clostridium pasteurianum LMG 3285

*Clostridium pasteurianum* was the second target species considered. Strain LMG 3282 was purchased from the Belgian Coordinated Collection of Microorganisms (BCCM/LMG, Gent, Belgium) in freeze-dried form, and it was revitalized in the optimal Reinforced Clostridium Medium (RCM) (Oxoid, Basingstoke, UK) for 7 days ± days in anaerobiosis at 37 °C. Spores were obtained following the protocol of Feng et al. (2010) [10]: 100 μL revitalized culture were inoculated on Potato Dextrose Agar (PDA) (Oxoid, Basingstoke, UK), modified with the addition of 0.75% (*w*/*v*) of calcium carbonate the modified agar medium and incubated at a suboptimal temperature of 30 °C in anaerobiosis for 30 days.

The presence of spores with respect to the vegetative cells was periodically checked using an optical microscope. When they were mainly present, 5 mL of sterile water were added to the agar plates, and the spores were collected after gently scraping. The spore solution was centrifuged and washed (5000× *g*—15 min—4 °C) with sterile water three times, resuspended in water/ethanol (50/50 *v*/*v*) and left at room temperature (approx. 22 °C) for one hour. Washing by centrifuge was repeated five times with sterile water, and then the spore solution was stored at 4 °C.

The sporal titer (Log cfu/mL) of the solution was evaluated by plate count. Before the count, the solution was thermal shocked at 80 °C for 10 min to induce the activation of the spores and the killing of any vegetative forms. The solution was decimally diluted in Ringer solution and dilutions plated in RCM agar medium. The plates were then incubated at 37 °C in anaerobic conditions for 48 h.

### 2.3. Design of Experiments (DOE)

The experiments were designed according to a mathematical model using MODDE Pro v12.0.1 software (MKS Umetrics, Umeå, Sweden), performing a total of 58 experiments for each strain. The model was planned around five quantitative factors with partial least square regression (PLS): pasteurization time, a_w_, pH, antimicrobial concentration and spore concentration. Natural antimicrobial was obtained by lactic acid fermentation of tomato by-products (peels and seeds), followed by liquid extraction, as reported in previous articles [6,7].

Briefly, tomato by-products were sterilized and inoculated with a pure LAB culture to a final concentration of 7 log10 cfu/mL. The inoculated tomato by-product was then incubated at 37 °C for 72 h, and the fermented substrate was lyophilized and subjected to extraction. The extract was freeze-dried to a powder and stored at −80 °C until use. The measured responses were the percentage of germination and the percentage of inactivation of the spores. Germination was defined as the percentage of spores that manage to germinate, after pasteurization. Inactivation was considered as the percentage of spores killed or damaged after heat treatment, therefore no longer able to germinate. The response surface model was elaborated with a Central Composite Full factorial design (CCF). This type of design requires setting three levels of analysis for each parameter: an average value, a maximum and a minimum value for a total of 29 experiments to be carried out in duplicate for each target strain. The values set for each parameter are summarized in Table 1.

The temperature was set at 90 °C and maintained constant to account for the pasteurization treatment generally used in the tomato processing plant.

The a_w_ ranges were chosen to reflect the values of the tomato-based products on the market (tomato paste a_w_ 0.94—tomato sauce 0.98). Also for pH, we choose a range that could reflect various tomato-based products. The three different levels of pasteurization time were chosen, starting from 10 min, which is the time usually set for pasteurization. The study of each model was performed in the optimal culture medium of the target microorganism. The pH of the solution was modified with Hydrochloric acid (HCl), while the a_w_ was modified by adding sodium chloride (Sigma-Aldrich, St. Louis, MO, USA) and glycerol (Sigma-Aldrich, St. Louis, MO, USA) at a known concentration. 

**Figure 1:**
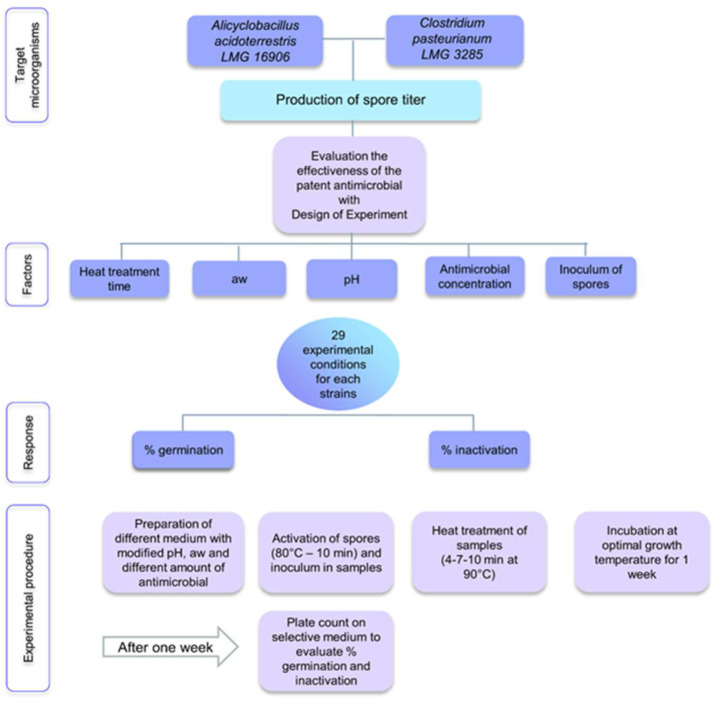
Experimental procedure of the study.

The experimental procedure is presented in Figure 1. The set of experiments was prepared by adding to the medium the antimicrobial, and then the spores of the target microorganisms after activation with thermal shock (80 °C—10 min), both at different concentrations according to the model. Once inoculated, the heat treatment at 90 °C was applied considering three different times according to the model (4–7–10 min). The samples were then incubated at the optimal temperature for one week, after which the percentage of germination and the percentage of inactivation of the spores were evaluated by plate count on a selective medium. 

### 2.4. Evaluation of Spore Germination and Inactivation

After a week of incubation, the concentration of vegetative cells and the number of spores that managed to germinate were evaluated by plate count. Samples were decimally diluted in Ringer and dilutions were plated on the optimal agar medium (AAM and RCM, for *A. acidoterrestris* and *C. pasteurianum* respectively). The percentage of germination was calculated on the basis of the initial inoculation of spores and the final concentration of cells, following the formula

(*n_t_*/*n*_0_) × 100

where *n_t_* is the vegetative cells germinate after one week of incubation and *n*_0_ is the initial inoculum of spores.

The samples were then analyzed to assess the inactivated spores. Samples were thermal shocked at 80 °C for 10 min to kill vegetative cells and activate the remaining spores, subsequently, plate count on the optimal medium was carried out. The percentage of inactivated spores was calculated starting from the initial concentration, following the formula

100 − (*n_t_*/*n*_0_) × 100

where *n_t_* is the spore load after one week of incubation and *n*_0_ is the initial inoculum of spores.

## 3. Results

### 3.1. Alicyclobacillus acidoterrestris LMG 16906

**Figure 2:**
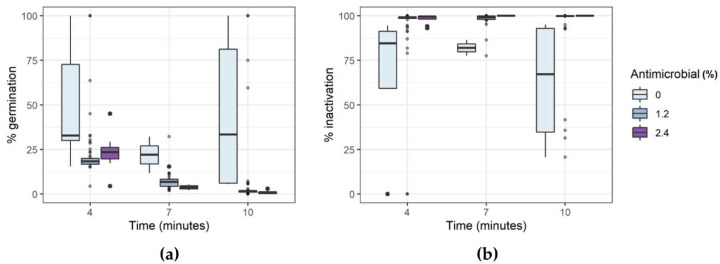
Percentage variation of germination (**a**) and inactivation (**b**) of *A. acidoterrestris* LMG 16906 spores after exposure to DoE conditions.

Exposure of *A. acidoterrestris* to the experimental conditions of the DoE reported in Table 1 had a different effect on germination and inactivation of the bacterial spores inoculated in the model system. As shown in Figure 1, exposure to an increasing thermal treatment had a variable effect on the germination (Figure 2a) and inactivation (Figure 2b) of the bacterial spores. The addition of the antimicrobial product, however, significantly reduced the germination of the spores and increased the inactivation, in all the selected conditions.

**Figure 3:**
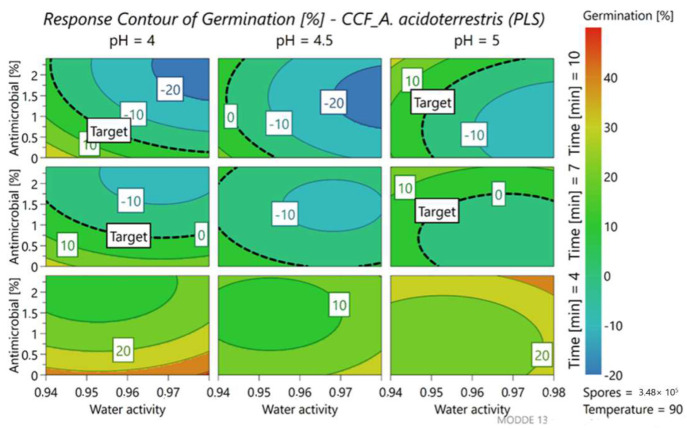
Contour plot representing the percent of germination of *A. acidoterrestris* LMG 16906, at different levels of parameters.

**Figure 4:**
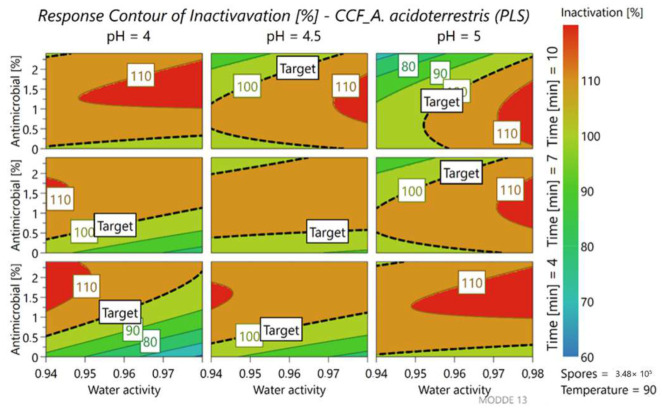
Contour plot representing the percent of spore inactivation of *A. acidoterrestris* LMG 16906, at different levels of parameters.

To better understand the variations of response, we kept the concentration of the inoculum fixed at the maximum level (5.5 Log cfu/mL). The accuracy of the model was described by the software using different statistical parameters such as R2 and Q2 that refer respectively to the model fit and the prediction precision. The value of these two parameters must be above 0.5. R2 results in 0.89 and 0.93 for germination and inactivation while the value of Q2 results in 0.80 and 0.86, respectively. Nine different contour plots, representing the response surface, were thus obtained (Figure 3 and Figure 4), which change depending on the duration of the heat treatment and the pH of the medium. The two axes correspond to the water activity of the medium and the percentage of antimicrobial added to the samples. To better visualize the area of interest, the target to reach, that is, the optimal response, was established at 0% for germination (blue-green zone) and 100% for inactivation (red-orange zone). In brief, an ideal treatment aims at reaching at least the target response (0% germination, represented by the dashed line in Figure 3) or even lower values, according to the color scale.

Looking at the graph corresponding to the percentage of germination (Figure 3), the response changed mainly as a function of the heat treatment time, pH values of the medium and quantity of antimicrobial added to the product.

Starting from the top of the graph, the first row shows samples treated for 10 min and highlights how the circle delimited by the target covers all the different ranges with different water activities. This means that in general, after one week of incubation, 0% germination is recorded when 10 min of heat treatment at 90 °C is set up. However, when the pH of the solution becomes less acidic (pH 5) the circle starts to restrict, but the percentage of germination remains within 10%. Interestingly, the addition of the antimicrobial leads to similar results (0% germination) even at shorter pasteurization times (7 min). The last line of the graphs corresponds to the shorter heat treatment time (4 min) and highlights that the total absence of germination is not possible but can be minimized by adding about 1% of antimicrobial.

Also, regarding spore inactivation (Figure 4) pH and treatment time are the parameters that affect the response, together with the a_w_ of the medium.

Observing the first column that corresponds to the samples inoculated in the media at pH 4, 100% inactivation was generally guaranteed for all a_w_ values but, as the a_w_ increases, the circle starts to narrow upwards. This highlights how the inactivation objective is guaranteed thanks to the addition of the antimicrobial. This condition also allows the treatment time to be reduced by up to 4 min.

### 3.2. Clostridium pasteurianum LMG 3285

**Figure 5:**
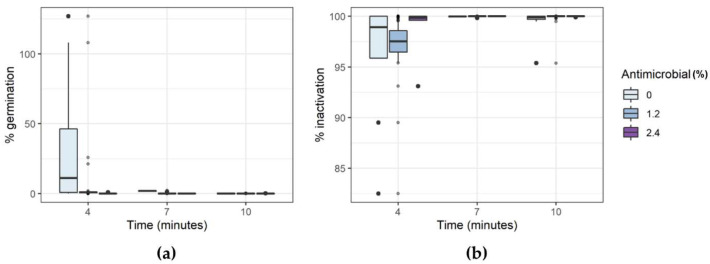
Percentage variation of germination (**a**) and inactivation (**b**) of *C. pasteurianum* LMG 3285 spores after exposure to DoE conditions.

Data collected for the selected *C. pasteurianum* strain upon exposure to the experimental conditions (Table 1) showed how the spores of this microorganism have a greater sensitivity to the selected treatments, compared to the strain of *A. acidoterrestris*. As shown in Figure 5, thermal treatment of 4 min in the absence of antimicrobial could reduce the germination and lead to a certain degree of spores’ inactivation, but with some variability, in relation to the physicochemical parameters of the model substrate (pH and a_w_). Even for this strain, the addition of antimicrobial to the model media led to a reduction in the sporal germination and an increased inactivation in all the tested conditions.

Also for this species, the analyzed samples were 58 and, keeping the concentration of the inoculum unchanged at the considered maximum level of 6 Log cfu/mL, the software created a response surface. In this case, R2 results in 0.68 and 0.58 for germination and inactivation while the value of Q2 results in 0.55 and 0.50, respectively.

**Figure 6:**
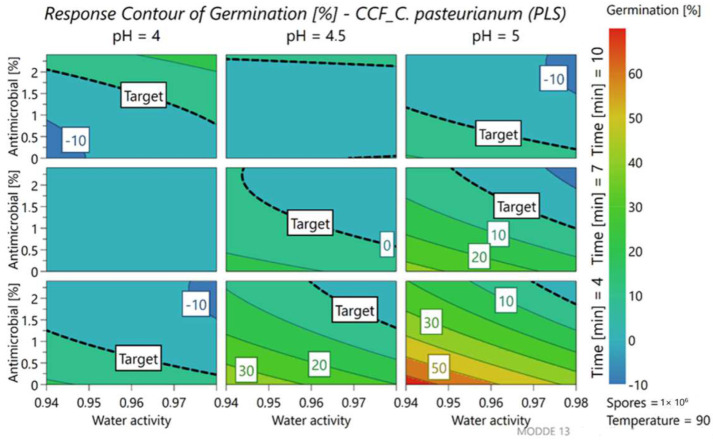
Contour plot representing the percent of germination of *C. pasteurianum* LMG 3285, at different levels of parameters.

*C. pasteurianum* exhibited similar behavior to *A. acidoterrestris*. The plots presented in Figure 6 show, also in this case, that the main factors influencing the percentage of germination were pH, heat treatment time and antimicrobial addition.

In general, at the lowest pH considered (pH 4), the percentage of germination remained low even with reduced treatment times (4 min). At pH 5 and with treatment times of less than 10 min, the target circle narrows but it would be still possible to obtain a low percentage of germination with the addition of the antimicrobial.

**Figure 7:**
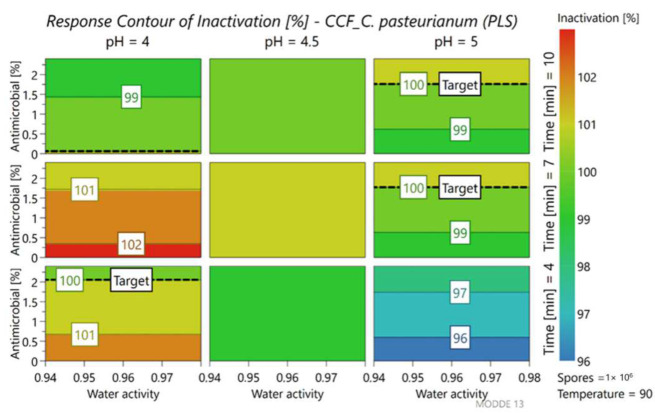
Contour plot representing the percent of spore inactivation of *C. pasteurianum* LMG 3285, at different levels of parameters.

Figure 7 highlights that the percentage of inactivated *C. pasteurianum* spores was very high and close to 100% in almost all cases. In fact, observing the box at the bottom right (best condition for growth with higher pH and low treatment times), even in the absence of antimicrobials, an inactivation of around 96% is obtained.

As for germination, the highest pH considered (pH 5) led to reduced effectiveness of heat treatment. However, the target of 100% inactivation was reached with the addition of antimicrobial extract.

## 4. Discussion

A current challenge for the food industry is to ensure the stability of the product, trying to increase its shelf life while minimizing the negative effects of heat treatments on organoleptic properties. Therefore, the addition of antimicrobials becomes often necessary, even if consumers are more attracted toward products labeled as “natural”, frowning on chemicals and preservatives in general [18]. In this debate, antimicrobials obtained from fermented plant substrates, like tomato by-products, could represent a valuable strategy for the food industry. Moreover, from a circular economy perspective, the application of this antimicrobial to a tomato-based product could close the loop of primary resource utilization and reduction of by-products. In this study, the effectiveness of such a natural antimicrobial to reduce the percentage of germination and the ability to inactivate spores of spoilage target microorganisms was evaluated. Results from the two DoE showed activity of the antimicrobial against spores of both species considered (*A. acidoterrestris* and *C. pasteurianum*) highlighting the pH, time of treatment and a_w_ as the parameters that most influence the response as reported by Silva et al. (1999) [14].

Bacterial endospores are characterized by their need for special requirements to initiate germination and outgrowth. Among these needs are optimum a_w_ values and nutrient availability [19]. As reported by Silva and Gibbs (2001) [17], the condition of the media strongly affects the heat resistance of the spores, and one of the most significant variables is the pH, which has a negative correlation with respect to the germination process, and it depends on the temperature used for thermal inactivation [14]. According to numerous studies, bacterial spores become more heat-resistant when water activity is reduced. In the present research, this trend referred to as germination was recorded for both the target species. Indeed, we observed that high a_w_ is associated with an increase in the percentage of germination. As described above, adding antimicrobials could lead to a shorter heat treatment to prevent the germination of *A. acidoterrestris* and *C. pasteurianum*. This finding is important as consumer demand for fresh, under-processed or unpasteurized foods has increased in recent decades due to their high levels of vitamin C, polyphenols and antioxidants deteriorating with treatments at high temperatures [20]. Various research studies in the literature have focused on the natural antibacterial activity of essential oils, plant extract and algae [21,22,23,24,25,26].

Moreover, the antibacterial activity of fermented extracts/substrate starting from by-products is poorly reported [27,28,29].

Extracts from fermented tomato by-products used in this study showed antimicrobial activity recently reported against food-borne pathogens both for in vitro and in situ studies [6,7]. In this study, its effectiveness toward different microbial targets, and in particular their spores, was also demonstrated. The tested antimicrobial reduced the ability of germination of spores and/or led to the inactivation of spores. The compounds responsible for this activity have not yet been identified but there are various hypotheses. The activity could derive from the compounds naturally present in substrates, such as polyphenols [30]. Furthermore, during fermentation, various organic acids or bacteriocins could be produced [31]. For example, the activity against spore-forming of nisin, a polypeptide produced by some strains of lactic acid bacteria has been reported [9,32].

## 5. Conclusions

The control of spoilage microorganisms, in particular spore-forming bacteria, represents a key element in the quality control of the processing industries. In the tomato industry, two of the major targets to be monitored during the production process are *Alcyclobacillus acidoterrestris* and *Clostridium pasteurianum* because they can deeply alter the final product.

In this study, thanks to the Design of Experiment (DoE) approach, it was possible to verify the influence of various factors on the germination and inactivation of the bacterial spores and to evaluate the process and product conditions that can guarantee the microbiological stability of the product, possibly reducing the heat treatment time, and therefore, the cost of the process. In addition, it was possible to study the effect of a new extract recently patented on the spores of microbial targets with the final aim to evaluate its use in tomato commercial products. 

The data obtained allowed the development of predictive models that can be implemented and managed for the design of new products or treatments. In our opinion, the creation of this model represents a useful tool for tomato processing companies but also for other companies that must keep the same targets under control (e.g., juice industries). Indeed, with the predictive function of the software it is possible to evaluate the different responses (germination, inactivation) according to the type of product to be considered (e.g., according to specific a_w_, pH). Future studies could be directed towards the validation of these models in commercial products, or the implementation of these data with product shelf life studies or with sensory data to verify the impact of the addition of the antimicrobial on the characteristics of the product.

## Figures and Tables

**Table 1:** Values of the quantitative factors.

Name	Units	Settings
Temperature	°C	90
Time	Min	4 to 10
Water activity		0.94 to 0.98
pH		4 to 5
Antimicrobial	%	0 to 2.4
Spores	cfu/mL	1× 10^4^ to 3.48 × 10^5^/10^6^

## Data Availability

Not applicable.
